# Homocysteine diminishes apolipoprotein A-I function and expression in patients with hypothyroidism: a cross-sectional study

**DOI:** 10.1186/s12944-016-0293-5

**Published:** 2016-07-26

**Authors:** Ning Yang, Zhi Yao, Li Miao, Jia Liu, Xia Gao, Yuan Xu, Guang Wang

**Affiliations:** Department of Endocrinology, Beijing Chaoyang Hospital, Capital Medical University, No. 8, Gongti South Road, Chaoyang district, Beijing 100020 People’s Republic of China

**Keywords:** Hypothyroidism, Homocysteine, Apolipoprotein A-I, High-density lipoprotein-cholesterol

## Abstract

**Background:**

Hypothyroidism (HO) can significantly impair lipid metabolism and increase cardiovascular disease risk. Hyperhomocysteinemia (HHcy) is an independent risk factor for cardiovascular disease. Our previous study demonstrated that HHcy significantly induced insulin resistance and impaired coronary artery endothelial function in patients with either hypertension or HO. In the present study, we studied whether plasma levels of high-density lipoprotein-cholesterol (HDL-C) and apolipoprotein A-I (Apo A-I) were altered in patients with HO, and if so, whether this change was mediated by HHcy.

**Methods:**

A total of 258 subjects were enrolled and divided into the following three groups: control group (*n* = 94), HO group (*n* = 73), and subclinical hypothyroidism (SHO) group (*n* = 91). Additionally, all groups were subdivided based on the subjects’ Hcy levels into HHcy (plasma Hcy level over 15 μmol/l) and normal Hcy subgroups. The plasma levels of lipid indexes were measured. Statistical analyses were performed to evaluate the correlations between groups.

**Results:**

The plasma Hcy levels were significantly higher in the HO group than in the SHO or control groups (all *p* < 0.05). Moreover, levels of Apo A-I and HDL-C were markedly reduced in the HHcy subgroup compared with the normal Hcy subgroup for patients with either HO (Apo A-I: *p* < 0.05; HDL-C: *p* < 0.01) or SHO (Apo A-I: *p* < 0.05; HDL-C: *p* < 0.01). In addition, the plasma Hcy levels were negatively correlated with levels of Apo A-I in all three groups (HO group: *r* = − 0.320, SHO group: *r* = − 0.337 and control group: *r* = − 0.317; all *p* < 0.01).

**Conclusions:**

Hcy levels were significantly increased in patients with HO or SHO. These increased Hcy levels may impair cardiovascular function via the inhibition of Apo A-1 expression and impairment of its antioxidant capacity. Our findings provide new insights into the pathogenesis of hypothyroidism-induced metabolic disorders.

## Background

Hypothyroidism (HO) and subclinical hypothyroidism (SHO), the two most common endocrine disorders, can induce metabolic dysfunction [1] and increase the risk of cardiovascular disease [2, 3]. In patients with HO or SHO, dyslipidemia may account for the high risk of cardiovascular disease, including elevated levels of total cholesterol (CHOL), low-density lipoprotein cholesterol (LDL-C) and triglycerides (TG) [4, 5]. The major role of high-density lipoprotein cholesterol (HDL-C) is reverse cholesterol transport, in which cholesterol from the peripheral tissue and vessel wall can be transported to the liver. HDL-C exerts anti-inflammatory, antioxidant, anticoagulant, and profibrinolytic activities that may further protect against cardiovascular disease [6]. The relationship between coronary artery disease and low levels of plasma HDL-C have been demonstrated in earlier studies [7, 8]. Apolipoprotein A-I (Apo A-I) is the major protein component of HDL-C and exerts anti-atherogenic effects via several mechanisms, including reverse cholesterol transport [9, 10]. Reduced HDL-C and Apo A-I levels increase the risk of cardiovascular disease.

Homocysteine (Hcy) levels are increased in patients with HO or SHO [11, 12], and Hcy is associated with the severity of lipid metabolism dysfunction. Hyperhomocysteinemia (HHcy) is an independent risk factor for atherosclerosis [13, 14]. Our previous study demonstrated that HHcy induced insulin resistance in patients with HO or SHO [15] and impaired coronary artery endothelial function in patients with hypertension or hypertriglyceridemia [16, 17]. Several studies have reported a negative association between levels of Hcy and Apo A-I and both cardiovascular events and atherosclerosis disease [18–20]. Thyroid hormones affect reverse cholesterol transport by increasing the activity of hepatic lipase and cholesteryl ester transfer protein (CETP), and these changes may result in HDL-C and Apo A- I being increased in HO. With increasing Hcy levels, however, some Hcy molecules are converted to homocysteine thiolactone (HcyT), which forms an isopeptide bond with the lysine residues of proteins; this process is known as N-homocysteinylation [21]. A recent study found that N-homocysteinylation of Apo A-I impairs its antioxidant ability [22]. However, associations between Hcy and Apo A- I levels have not been well characterized. In this present study, we tested for potential associations between levels of Hcy and Apo A-I that could help explain the increased risk of arteriosclerotic coronary artery disease associated with HO and SHO.

## Methods

### Subjects

This study initially enrolled 236 outpatients and was conducted from January to December 2013 at the Department of Endocrinology in Beijing Chaoyang Hospital. HO is a thyroid hormone deficiency and can develop as a primary disease of the thyroid gland itself. An elevated thyrotropin (TSH) level, usually above 10 μIU/ml, along with a subnormal free thyroxine (FT4) level, characterizes overt HO [23]. SHO is defined as a serum TSH concentration above the statistically defined upper limit of the reference range and when serum FT4 and free triiodothyronine (FT3) concentrations are within the normal reference ranges [24, 25]. This designation is only applicable when thyroid function has been stable for several weeks, the hypothalamic–pituitary–thyroid axis is normal, and there is no recent or ongoing severe illness. Exclusion criteria were as follows: patients with cardiovascular disease, hypertension, diabetes mellitus or impaired glucose tolerance, renal diseases or other endocrine diseases. Therefore, 72 patients were excluded. The final study cohort consisted of 164 patients among those individuals who were initially enrolled, including 73 and 91 patients with HO and SHO, respectively. None of the patients received any treatment. The control group included 94 normal, non-HO volunteers who were seeking routine medical care at the physical examination centre of Beijing Chaoyang Hospital.

HHcy was defined as a plasma Hcy level greater than 15 μmol/l [17]. Based on the presence of HHcy, each group was subdivided into two subgroups. The normal reference value of Hcy was less than 15 μmol/L. In the HO group, the patients with a plasma level of Hcy > 15 μmol/l were termed the H-HO group (*n* = 36), and the other patients made up the N-HO group (*n* = 37). Similarly, we divided the SHO group into the H-SHO (*n* = 32) and N-SHO (*n* = 59) groups, and the control group was split into the H-control (*n* = 18) and N-control (*n* = 76) groups.

### Sample collection

Basic demographic data (i.e., age, sex, body height and weight) were collected from each patient. Subjects wore only underwear for height and weight measurements, which were assessed to the nearest 0.5 cm and 0.1 kg, respectively, by a well-trained examiner. Body mass index (BMI) was calculated as follows: bodyweight(kg)/[patient height(m)]^2^. After overnight fasting, blood samples were collected from the peripheral vein of each patient and subjected to a routine analysis that consisted of Hcy, CHOL, HDL-C,LDL-C, TG, Apo A-I, Apolipoprotein B (Apo B), FT3, FT4, and TSH measurements.

### Measurements of plasma levels of Hcy

Plasma Hcy concentrations were determined using an enzymatic cycling assay-based quantitative method using kits from Baiding Biotech (Beijing, China) according to the manufacturer’s instructions. The normal reference value was less than 15 μmol/l.

### Measurements of blood lipid and thyroid function indexes

Levels of CHOL, HDL-C, LDL-C, TG, Apo A-I, and Apo B were determined using a Dade-Behring Dimension RXL Autoanalyser (Dade Behring Diagnostics, Marburg, Germany). Reference intervals for CHOL, HDL-C, LDL-C, TG, Apo A-I and Apo B were 3.62–5.70 mmol/l, 1.03–1.55 mmol/l, 1.81–3.36 mmol/l, 0.56–2.26 mmol/l, 1.00–1.70 g/l, and 0.40–1.20 g/l, respectively.

Levels of FT3, FT4 and TSH were determined by an electrochemiluminescence immunoassay (ECLIA) using an Abbott Architect I2000 (Abbott Diagnostics, Abbott Park, IL, USA). Reference intervals for FT3, FT4, and TSH were 1.71–3.71 pg/ml, 0.7–1.48 ng/dl and 0.35–4.94 μIU/ml, respectively.

### Statistical analysis

All statistical analyses were performed using the Statistical Package for the Social Sciences software package (version 17.0, SPSS Inc., Chicago, IL, USA) to identify significant effects between patient groups and corresponding controls. Because Hcy and TG did not follow a normal distribution, comparisons between groups were performed using Mann–Whitney U or Kruskal–Wallis H tests. Values are expressed as medians (25th and 75th percentiles), and qualitative data are expressed as means ± standard deviations (SDs). Comparisons among groups were performed using independent-samples or one-way ANOVA. Spearman’s rank correlation was used to test for associations between Hcy and other variables. All tests were two-tailed, and *p*-values less than 0.05 were considered to be significant.

## Results

### Clinical characteristics of the study subjects

**Fig. 1:**
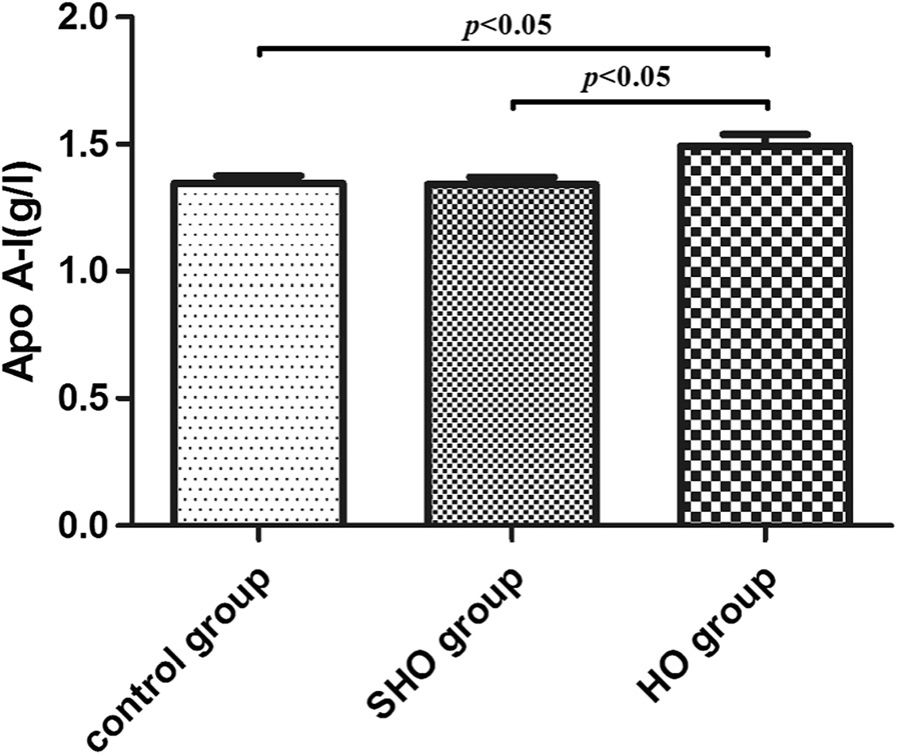
Plasma levels of Apo A-I in the study subjects. *n* = 94 in the control group; *n* = 91 in the SHO group and *n* = 73 in the HO group

**Fig. 2:**
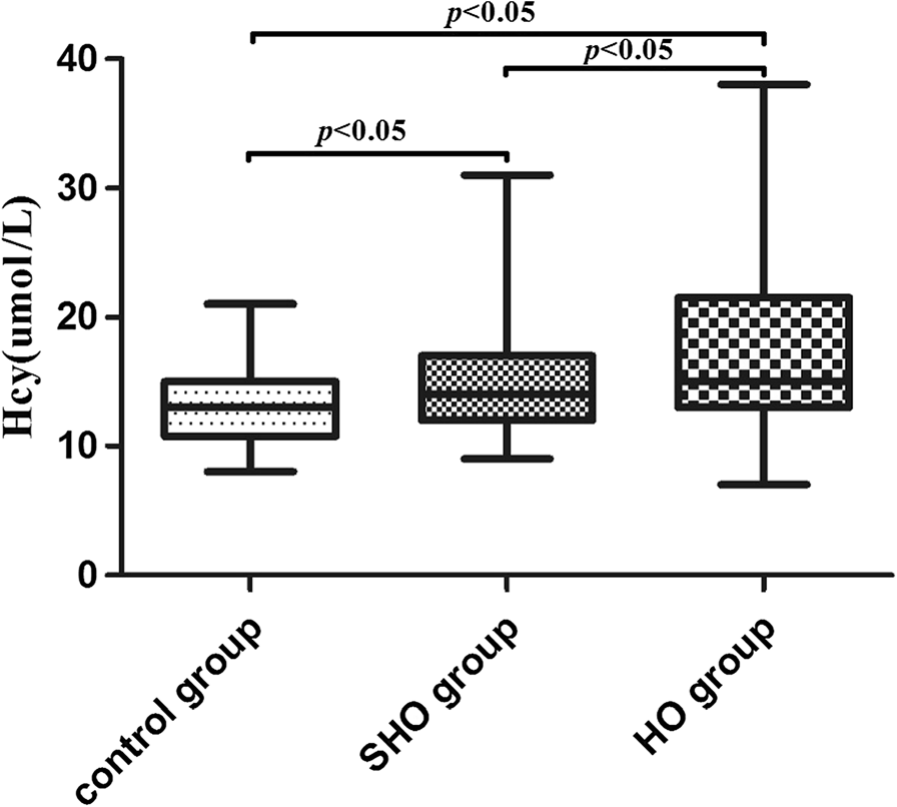
Plasma levels of Hcy in the study subjects. *n* = 94 in the control group; *n* = 91 in the SHO group and *n* = 73 in the HO group

The clinical characteristics and laboratory test findings of subjects are summarized in Table 1. The incidence of HO or SHO in females was significantly higher than that in males. The CHOL, HDL-C, LDL-C, Apo A-I and Apo B values in the HO group were significantly higher than those in the SHO and control groups; no differences were detected between the SHO and control groups [CHOL: 5.98 ± 1.67 vs. 5.09 ± 1.21 and 5.00 ± 1.00 mmol/l; HDL-C: 1.62 ± 0.40 vs. 1.47 ± 0.33 and 1.47 ± 0.30 mmol/l; LDL-C:  3.47± 1.21 vs. 3.03 ± 0.95 and 2.89 ± 0.81 mmol/l; Apo A-I: 1.49 ± 0.38 vs. 1.34 ± 0.28 and 1.35 ± 0.27 g/l (Fig. 1); Apo B: 1.01 ± 0.34 vs. 0.92 ± 0.26 and 0.89 ± 0.24 g/l; respectively; all *p* < 0.05]. A significant increase in the Hcy levels was observed in patients with HO compared with those in the SHO and control groups [15.00 (13.00–21.50) vs. 14.00 (12.00–17.00) vs. 13.00 (10.75–15.00) μmol/l, respectively, all *p* < 0.05] (Fig. 2). No significant difference was identified among the three groups for age, BMI or TG levels.

**Table 1:** Characteristics and laboratory test findings for each group involved in this study

Variables	Control group (*n* = 94)	SHO (*n* = 91)	HO (*n* = 73)	*p*-value
Sex, M/F	10/84	9/82	8/65	0.973
Age, years	46.78 ± 11.22	45.96 ± 13.97	43.07 ± 13.55	0.077
BMI, km/m^2^	24.15 ± 3.25	24.57 ± 3.31	24.63 ± 3.19	0.990
CHOL, mmol/l	5.00 ± 1.00	5.09 ± 1.21	5.98 ± 1.67†‡	<0.001**
HDL-C, mmol/l	1.47 ± 0.30	1.47 ± 0.33	1.62 ± 0.40†‡	0.027*
LDL-C, mmol/l	2.89 ± 0.81	3.03 ± 0.95	3.47 ± 1.21†‡	0.001**
TG, mmol/l	1.12 (0.79–1.69)	1.16 (0.78–1.78)	1.28 (0.88–1.76)	0.708
Apo A-I, g/l	1.35 ± 0.27	1.34 ± 0.28	1.49 ± 0.38†‡	0.005**
Apo B, g/l	0.89 ± 0.24	0.92 ± 0.26	1.01 ± 0.34†‡	0.007**
Hcy, μmol/l	13.00 (10.75–15.00)	14.00 (12.00–17.00)†	15.00 (13.00–21.50)†‡	<0.001**

Summary of the clinical characteristics and laboratory test results of the study participants (94 controls, 91 patients with SHO and 73 patients with HO). The data are expressed as means ± SDs unless otherwise indicated. TG and Hcy levels are presented as medians (25th and 75th percentiles). *BMI* body mass index, *CHOL* total cholesterol, *HDL*-*C* high-density lipoprotein cholesterol, *LDL*-*C* low-density lipoprotein cholesterol, *TG* triglycerides, *Apo A*-*I* apolipoprotein A-I, *Apo B* apolipoprotein B, *Hcy* homocysteine. Comparisons among groups were performed using aone-way ANOVA test. Because TG and Hcy levels did not follow a normal distribution, comparisons between groups were performed using the Mann–Whitney U test or Kruskal–Wallis H test. *p < 0.05, **p < 0.01, significantly different among the three groups; †*p* < 0.05, significantly different compared with the control group; ‡*p* < 0.05, significantly different compared with the SHO group

### Subgroup analysis of study participants

**Fig. 3:**
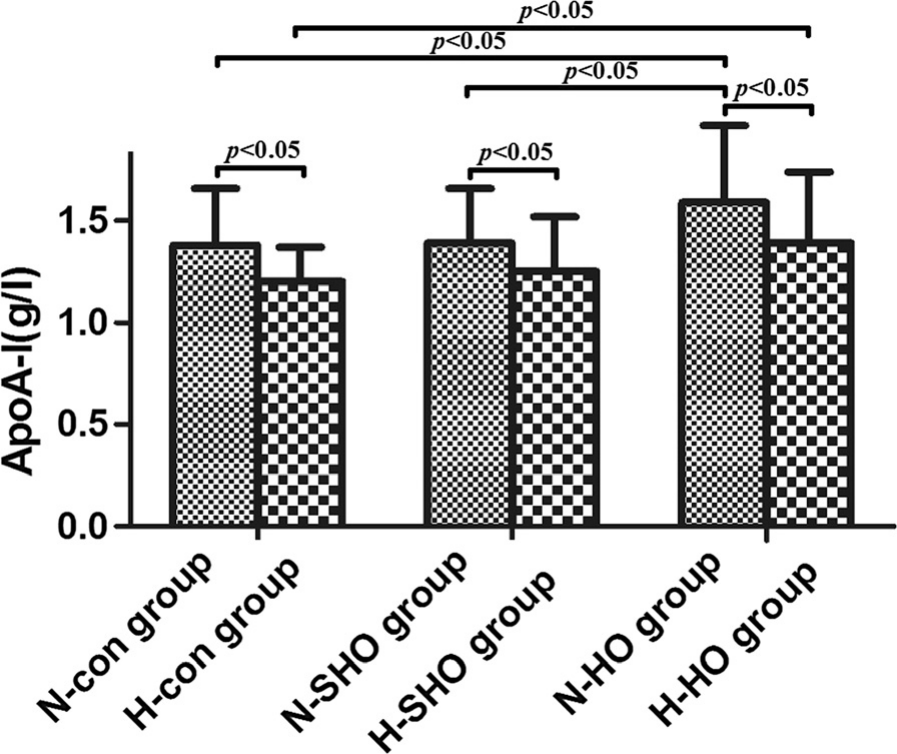
Apo A-I values in the study subgroups. *n* = 76 in the N-control group (control subjects with a plasma level of Hcy ≤ 15 μmol/l); *n* = 18 in the H-control group (control subjects with a plasma level of Hcy > 15 μmol/l); *n* = 59 in the N-SHO group (patients with SHO and a plasma level of Hcy ≤ 15 μmol/l); *n* = 32 in the H-SHO group (patients with SHO and a plasma level of Hcy > 15 μmol/l); *n* = 37 in the N-HO group (patients with HO and a plasma level of Hcy ≤ 15 μmol/l); *n* = 36 in the H-HO group (patients with HO and a plasma level of Hcy > 15 μmol/l)

The characteristics and blood lipid indexes of the patient subgroups are summarized in Table 2. Levels of CHOL were higher in the H-HO group than in the H-SHO and H-control groups; no difference was detected between the H-SHO and H-control groups (6.24 ± 2.06 vs. 5.26 ± 1.69 and 5.03 ± 0.90 mmol/l, respectively, *p* < 0.05). Levels of Apo A-I were higher in the H-HO than in the H-control group (1.39 ± 0.35 vs. 1.20 ± 0.17 mmol/l, *p* < 0.05); no significant differences associated with HHcy were detected for the other lipid indexes among the three patient subgroups. The levels of CHOL, HDL-C, LDL-C and Apo A-I in the N-HO group were significantly higher than those in the N-SHO and N-control groups; no significant differences were detected between the N-SHO and N-control groups [CHOL: 5.73 ± 1.15 vs. 4.99 ± 0.85 and 4.99 ± 1.03 mmol/l; HDL-C: 1.79 ± 0.34 vs. 1.54 ± 0.32 and 1.48 ± 0.31 mmol/l; LDL-C:3.36 ± 0.94 vs. 2.91 ± 0.72 and 2.87 ± 0.82 mmol/l; Apo A-I: 1.59 ± 0.38 vs. 1.39 ± 0.27 and 1.38 ± 0.28 g/l (Fig. 3); respectively; all *p* < 0.05]. The levels of HDL-C and Apo A-I were lower in the H-HO group than in the N-HO group (HDL-C:1.44 ± 0.37 vs. 1.79 ± 0.34 mmol/l, respectively, *p* < 0.01; Apo A-I: 1.39 ± 0.35 vs. 1.59 ± 0.38 g/l, respectively, *p* < 0.05). The levels of HDL-C and Apo A-I were lower in the H-SHO than in the N-SHO group (HDL-C: 1.35 ± 0.31 vs. 1.54 ± 0.32 mmol/l, respectively, *p* < 0.01; Apo A-I: 1.25 ± 0.27 vs. 1.39 ± 0.27 g/l, respectively, *p* < 0.05). Finally, the levels of Apo A-I were lower in the H-control than in the N-control group (1.20 ± 0.17 vs. 1.38 ± 0.28 g/l, respectively, *p* < 0.05).

**Table 2:** Comparison of characteristics and the laboratory test findings for the subgroups of each group

Parameters	Control group		SHO group		HO group	
	N-control group (*n* = 76)	H-control group (*n* = 18)	N-SHO group (*n* = 59)	H-SHO group (*n* = 32)	N-HO group (*n* = 37)	H-HO group (*n* = 36)
Sex, M/F	6/70	4/14	6/53	3/29	1/36	7/29
Age, years	46.42 ± 11.21	48.28 ± 11.46	45.41 ± 13.98	46.97 ± 14.10	41.73 ± 10.48	44.44 ± 16.16
BMI, km/m^2^	23.89 ± 3.09	25.21 ± 3.77	24.22 ± 3.35	25.19 ± 3.19	23.96 ± 3.32	25.32 ± 2.94
CHOL, mmol/l	4.99 ± 1.03	5.03 ± 0.90	4.99 ± 0.85	5.26 ± 1.69	5.73 ± 1.15^cd^	6.24 ± 2.06^ab^
HDL-C, mmol/l	1.48 ± 0.31	1.41 ± 0.25	1.54 ± 0.32	1.35 ± 0.31**	1.79 ± 0.34 ^cd^	1.44 ± 0.37**
LDL-C, mmol/l	2.87 ± 0.82	2.97 ± 0.76	2.91 ± 0.72	3.25 ± 1.26	3.36 ± 0.94 ^cd^	3.58 ± 1.43
TG, mmol/l	1.18 (0.78–1.72)	1.11 (0.94–1.55)	1.20 (0.77–1.79)	1.10 (0.79–1.64)	1.03 (0.83–1.63)	1.39 (0.92–2.51)
ApoA-I, g/l	1.38 ± 0.28	1.20 ± 0.17*	1.39 ± 0.27	1.25 ± 0.27*	1.59 ± 0.38 ^cd^	1.39 ± 0.35^a^*
ApoB, g/l	0.88 ± 0.25	0.89 ± 0.21	0.89 ± 0.22	0.96 ± 0.32	0.99 ± 0.29 ^c^	1.03 ± 0.39

To investigate the association among patient characteristics, blood lipid indexes and HHcy, we subdivided each group of patients into two groups according to the plasma Hcy levels. In the HO group, patients with a plasma level of Hcy > 15 μmol/l were termed the H-HO group (*n* = 36), while other patients were included in the N-HO group (*n* = 37). Similarly, we subdivided the SHO group into H-SHO (*n* = 32) and N-SHO (*n* = 59) subgroups and subdivided the control group into the H-control (*n* = 18) and N-control (*n* = 76) groups. The data are expressed as the means ± SDs unless otherwise indicated. TG is presented as medians (25th and 75th percentiles). Comparisons among groups were performed with independent-samples or one-way ANOVA tests. Because TG did not follow a normal distribution, between-group comparisons were performed using the Mann–Whitney U test or Kruskal–Wallis H test. *p < 0.05, **p < 0.01 indicate a significant difference between two subgroups; ^a^ *p* < 0.05, significantly different compared with the H-control group; ^b^ *p* < 0.05, significantly different compared with the H-SHO group; ^c^ *p* < 0.05, significantly different compared with the N-control group; ^d^ *p* < 0.05, significantly different compared with the N-SHO group

### Correlation between plasma levels of Hcy and both thyroid function and blood lipid indexes

Table 3 shows that Hcy was negatively correlated with the levels of FT3 and FT4 (*r* = −0.543 and −0.504, *p* < 0.01, respectively) in the HO group and positively correlated with TSH levels in the HO (*r* = 0.461, *p* < 0.01) and SHO (*r* = 0.264, *p* < 0.05) groups. The plasma Hcy levels were negatively correlated with HDL-C in both the HO and SHO groups (*r* = −0.375 and −0.356, all *p* < 0.01, respectively). Additionally, the Hcy levels were negatively correlated with Apo A- I in the HO, SHO and control groups (*r* = −0.320, −0.337 and −0.317; all *p* < 0.01, respectively).

**Table 3:** Correlations among plasma Hcy levels, thyroid function, and blood lipid indexes

	Control group (*n* = 94)		SHO (*n* = 91)		HO (*n* = 73)	
	*r*	*p*-*value*	*r*	*p*-*value*	*r*	*p*-*value*
Age, years	0.101	0.331	0.114	0.284	0.048	0.685
BMI, km/m^2^	0.139	0.180	−0.090	0.398	0.121	0.309
FT3, pg/ml	−0.178	0.087	0.125	0.239	−0.543	<0.001**
FT4, ng/dl	−0.102	0.327	−0.184	0.080	−0.504	<0.001**
TSH, μIU/ml	−0.029	0.781	0.264	0.011*	0.461	<0.001**
CHOL, mmol/l	0.097	0.352	0.093	0.383	0.128	0.282
HDL-C, mmol/l	−0.178	0.085	−0.356	0.001**	−0.375	0.001**
LDL-C, mmol/l	0.082	0.434	0.204	0.052	0.046	0.697
TG, mmol/l	−0.041	0.693	0.039	0.716	0.103	0.387
Apo A-I, g/l	−0.317	0.002**	−0.337	0.001**	−0.320	0.006**
Apo B, g/l	0.073	0.482	0.185	0.080	0.002	0.990

To investigate the correlation among plasma Hcy levels, blood lipids and thyroid function indexes in controls and patients with HO or SHO, we used Spearman’s rank correlation to assess these potential associations. *FT3* free tri-iodothyronine, *FT4* free thyroxine, *TSH* thyrotropin; *p < 0.05, **p < 0.01

## Discussion

HO and SHO are both associated with atherosclerosis [2, 3]. A major factor that contributes to this condition is dyslipidemia, which is observed in these patients [4, 5]. The relationship between coronary artery disease and low levels of plasma HDL-C has been demonstrated in earlier, prospective studies [7, 8]. Apo A-I is a major protein component of HDL-C and may exert anti-atherogenic effects through several mechanisms. Reduced levels HDL-C and Apo A-I increase the risk of cardiovascular disease. However, some studies and our present findings indicate that HDL-C and Apo A-I levels are increased in patients with HO or SHO and did not show an effect on anti-atherosclerotic function. HHcy is an independent risk factor for cardiovascular disease [13, 14]. HHcy increases cardiovascular disease via various mechanisms, including endothelial dysfunction, oxidative stress, endoplasmic reticulum stress, smooth muscle cell proliferation and platelet aggregation [26–29]. Our previous study demonstrated that HHcy induced insulin resistance in patients with HO or SHO [15] and impaired coronary artery endothelial function in patients with hypertension or hypertriglyceridemia [16, 17]. Some studies have shown that Hcy levels are increased in hypothyroid patients [11, 12]. Our previous study demonstrated that HHcy might contribute to atherogenesis by enhancing the responsiveness of monocytes to inflammatory stimuli and thereby promoting insulin resistance via the induction of endoplasmic reticulum stress in adipose tissue along with impairing coronary artery endothelial function [16, 17]. HHcy can inhibit reverse cholesterol transport by reducing circulating HDL via the inhibition of Apo A-I protein synthesis, resulting in an increased risk of atherosclerosis [30, 31]. Concurrently, other studies reported that HHcy impaired the antioxidant ability of Apo A-I and HDL-C [22]. In this present study, we provide for the first time evidence for a negative correlation between Hcy and Apo A-I in patients with HO or SHO.

We observed that subjects in the control and SHO groups had lower levels of CHOL, HDL-C, LDL-C, Apo A-I and Apo B than individuals in the HO group. By altering lipid metabolism, HO accelerates the process of atherogenesis and increases cardiovascular risk. Overt HO is characterized by hypercholesterolemia and a marked increase in LDL-C and Apo B because of decreased fractional clearance of LDL-C, which is a consequence of fewer LDL-C receptors in the liver. Apo A-I, a major protein component of HDL, is a major contributor to the anti-atherosclerotic effects. Levels of HDL-C are elevated in HO because of decreased CETP and hepatic lipase activity, which are both enzymes regulated by thyroid hormones [32]. Increased levels of CHOL, LDL-C and Apo B are linked to atherosclerosis. Hcy was significantly higher in the HO group than in the SHO and control groups. Our findings are consistent with those of previous studies [11, 12]. Hcy is a sulfur-containing amino acid that is formed during methionine metabolism. Intensive studies have identified HHcy as an independent risk factor for atherosclerosis [13, 14]. Our previous study demonstrated that HHcy may contribute to atherogenesis by enhancing the responsiveness of monocytes to inflammatory stimuli and promoting leukocyte recruitment into atherosclerotic plaques [33]. A portion of Hcy is converted to HcyT by methionyl-tRNA synthetase. Indeed, levels of plasma HcyT are increased in humans with HHcy caused by mutations in the cystathionine β-synthase or 5,10-methylene-tetrahydrofolate reductase genes [21]. HcyT, a cyclic thioester, forms an isopeptide bond with lysine residues of proteins, which is known as *N*-homocysteinylation. The *N*-homocysteinylation of proteins can lead to the loss or modulation of protein function. In addition, *N*-homocysteinylated proteins may cause atherosclerosis via cell death, inflammation, and adaptive immune responses. *N*-homocysteinylated Apo A-I is present in normal human serum at a proportion of 1.0–7.4 % of *N*-Hcy-Apo A-I among total Apo A-I [11]. With increasing levels of Hcy in patients with HO or SHO, levels of *N*-Hcy-Apo A-I may be increased. A recent study found that N-Hcy-Apo A-I promotes LDL-C oxidation, and HcyT-treated HDL-C also loses its antioxidant activity. Therefore, increased levels of Apo A-I and HDL-C do not affect antioxidant ability because N-homocysteinylation of Apo A-I impairs this function [22]. Dyslipidemia and HHcy may both be associated with endothelial dysfunction and cardiovascular disease.

To investigate the association between HHcy and blood lipid indexes, we performed a subgroup analysis. Each patient group was subdivided into two subgroups according to the plasma Hcy levels. Similar to other studies, the levels of CHOL, HDL-C, LDL-C and Apo A-I in the HO group were higher than those in the SHO and control groups for patients with normal plasma levels of Hcy. However, among the three hyperhomocysteinemic subgroups, the differences in HDL-C and Apo A-I values were not significant, and Apo A-I only showed a difference between the H-HO and H-control groups, with lower levels in the latter group. Levels of HDL-C and Apo A-I were significantly lower in the hyperhomocysteinemic subgroups compared with the subgroups of patients with HO or SHO and normal plasma Hcy levels. Apo A-I and HDL-C can reduce the risk of cardiovascular disease by promoting reverse cholesterol transport and modulating inflammation [9, 10]. Hcy can inhibit reverse cholesterol transport by reducing circulating HDL-C via the inhibition of the expression and protein synthesis of Apo A-I and the enhancement of HDL-C clearance in an animal model [30, 31]. This finding suggests that Hcy inhibited the expression of Apo A-I and HDL-C in patients with HO or SHO, which could increase the risk of cardiovascular disease.

Our study showed that Hcy was negatively correlated with levels of FT3 and FT4 in the HO group and positively correlated with levels of TSH in the SHO and HO groups. Several other studies have proposed that HO reduces hepatic levels of the enzymes involved in Hcy metabolism. This hypothesis has been supported by the finding that HO and SHO are associated with alterations in plasma levels of Hcy [34, 35]. We observed that plasma levels of Hcy were negatively correlated with those of HDL-C in the HO and SHO groups and were negatively correlated with levels of Apo A-I among the three groups. With an increased level of Hcy, there was a concurrent reduction in the levels of HDL-C and Apo A-I. Similar correlations have been reported in other diseases [30, 31]. To the best of our knowledge, we have established for the first time that plasma Hcy levels show a significant, negative correlation with Apo A-I concentrations in patients with HO or SHO. Our findings suggest that HHcy impairs the antioxidant ability of Apo A-I and inhibits the expression of Apo A-I and HDL-C; these events are associated with an increased risk of cardiovascular disease in patients with HO or SHO.

## Conclusions

Plasma Hcy levels were significantly increased in patients with HO or SHO. These increased levels of Hcy may impair cardiovascular function by impairing the antioxidant ability of Apo A-I and inhibiting the expression of Apo A-I and HDL-C. Our findings suggest that HHcy may increase cardiovascular risk via multiple effects, especially through a mechanism that can induce dyslipidemia in patients with HO and SHO. Thus, our findings provide new insights into the pathogenesis of HO- and SHO-induced metabolic disorders.

## Abbreviations

Apo A-I, apolipoprotein A-I; Apo B, apolipoprotein B; BMI, body mass index; CETP, cholesteryl ester transfer protein; CHOL, total cholesterol; FT3, free triiodothyronine; FT4, free thyroxine; Hcy, homocysteine; HcyT, homocysteine thiolactone; HDL-C, high-density lipoprotein cholesterol; HHcy, hyperhomocysteinemia; HO, hypothyroidism; LDL-C, low-density lipoprotein cholesterol; SD, standard deviation; SHO, subclinical hypothyroidism; TG, triglyceride; TSH, thyrotropin
